# The impact of nasogastric tube feeding on Drive for Thinness and Body Dissatisfaction in children and adolescents with Anorexia Nervosa

**DOI:** 10.1192/j.eurpsy.2022.975

**Published:** 2022-09-01

**Authors:** J. Pruccoli, M. Pelusi, G. Romagnoli, A. Parmeggiani

**Affiliations:** University of Bologna, Child Neurology And Psychiatry, Bologna, Italy

**Keywords:** Anorexia nervosa, children and adolescents, Nasogastric tube feeding

## Abstract

**Introduction:**

The use of nasogastric-tube feeding (NGT) in the treatment of Anorexia Nervosa (AN) in children and adolescents is recommended by current guidelines. Nonetheless, the literature lacks studies assessing prognostic factors for modifications of AN-specific psychopathology treated with NGT.

**Objectives:**

To assess potential prognostic affecting improvement in AN-specific psychopathology in children and adolescents hospitalized for AN, treated with NGT.

**Methods:**

Retrospective study assessing young inpatients with AN, treated with NGT. Considered outcomes (admission vs discharge) were AN-specific psychopathology (Eating Disorder Inventory-3 (EDI-3): Drive for Thinness (DT); Body Dissatisfaction (BD); Eating-Disorders Risk (EDRC)) and body-mass index (BMI). Considered potential predictors were demographics, duration of untreated illness (DUI), severity (admission BMI), diagnoses, early vs late (0-7 vs 8+ days after admission) start of NGT, drugs). Models for specific contributions of predictors related to outcomes were assessed with analysis of covariance (ANCOVA).

**Results:**

Fifty-three inpatients (F=53, mean age 15.1±2.0 years) were enrolled. Both higher DT (F(1,22)=15.07, p<0.001) and BD improvement (F(1,22)=7.73, p=0.011) were predicted by lower admission BMI. Higher BMI improvement was predicted by lower admission BMI (F(1,47)=10.39, p<0.001) and age (F(1.47)=6.12, p=0.011. AN subtypes, comorbidities, antidepressants, and different antipsychotics did not predict any outcome.

**Conclusions:**

In this study, greater improvement in AN-specific psychopathology (DT and BD) and weight in patients treated with NGT was predicted by lower admission BMI. These results suggest that young patients with greater severity may highly benefit from NGT. These findings, if confirmed in wider and controlled samples, could help in optimizing the treatment with NGT in young inpatients with AN.

**Disclosure:**

No significant relationships.

